# Carbapenem Resistance in *Klebsiella pneumoniae* Not Detected by Automated Susceptibility Testing

**DOI:** 10.3201/eid1208.060291

**Published:** 2006-08

**Authors:** Fred C. Tenover, Rajinder K. Kalsi, Portia P. Williams, Roberta B. Carey, Sheila Stocker, David Lonsway, J. Kamile Rasheed, James W. Biddle, John E. McGowan, Bruce Hanna

**Affiliations:** * Centers for Disease Control and Prevention, Atlanta, Georgia, USA;; † Bellevue Hospital, New York, New York, USA;; ‡ Rollins School of Public Heath, Emory University, Atlanta, Georgia, USA

**Keywords:** carbapenem, imipenem, meropenem, susceptibility testing, Klebsiella, beta-lactamase, carbapenemase, research

## Abstract

Detecting β-lactamase–mediated carbapenem resistance among *Klebsiella pneumoniae* isolates and other *Enterobacteriaceae* is an emerging problem. In this study, 15 *bla*_KPC_-positive *Klebsiella pneumoniae* that showed discrepant results for imipenem and meropenem from 4 New York City hospitals were characterized by isoelectric focusing; broth microdilution (BMD); disk diffusion (DD); and MicroScan, Phoenix, Sensititre, VITEK, and VITEK 2 automated systems. All 15 isolates were either intermediate or resistant to imipenem and meropenem by BMD; 1 was susceptible to imipenem by DD. MicroScan and Phoenix reported 1 (6.7%) and 2 (13.3%) isolates, respectively, as imipenem susceptible. VITEK and VITEK 2 reported 10 (67%) and 5 (33%) isolates, respectively, as imipenem susceptible. By Sensititre, 13 (87%) isolates were susceptible to imipenem, and 12 (80%) were susceptible to meropenem. The VITEK 2 Advanced Expert System changed 2 imipenem MIC results from >16 μg/mL to <2 μg/mL but kept the interpretation as resistant. The recognition of carbapenem-resistant *K. pneumoniae* continues to challenge automated susceptibility systems.

Carbapenem resistance among the *Enterobacteriaceae* is emerging in the United States, particularly on the East Coast (1–6). Resistance to the most widely used carbapenems, i.e., imipenem and meropenem, can be mediated by a variety of mechanisms, including β-lactamases, porin changes, and changes in penicillin-binding proteins (1,7,8). KPC enzymes are among the most common β-lactamases mediating carbapenem resistance among isolates of *Enterobacteriaceae* (1–6). KPC enzymes are class A β-lactamases that mediate resistance to extended-spectrum cephalosporins in addition to carbapenems; these β-lactamases are usually plasmid encoded.

Clinical microbiology laboratories have often found it difficult to achieve accurate susceptibility testing results for carbapenem drugs. Early studies documented false resistance to imipenem due to degradation of the drug (9); later studies with the VITEK system (bioMérieux, Durham, NC, USA) demonstrated false resistance, specifically with *Proteus mirabilis* (10). Several recent proficiency testing studies have shown problems of both false resistance and false susceptibility with imipenem and meropenem among a variety of enteric species (11,12). Even quality control measures fail to detect all false resistance problems (13).

Yigit and colleagues described the KPC-1 β-lactamase in 2001 (1). The β-lactamase was identified in an imipenem-resistant isolate of *Klebsiella pneumoniae* from the United States. Subsequently, 3 additional KPC-type β-lactamases have been described from *Salmonella*, *K. oxytoca*, *Enterobacter cloacae*, and other *K. pneumoniae*; these differ in amino acid sequence from each other, typically by 1 or 2 amino acids (2–6). Bratu and colleagues reported false-susceptible results for *K. pneumoniae* isolates with the MicroScan WalkAway system (Dade MicroScan, Inc., West Sacramento, CA, USA), which were attributed in part to low inoculum size (14). Similar problems with false-susceptible results were noted with the VITEK system (15). The goal of this study was to conduct a rapid assessment of currently available antimicrobial susceptibility testing methods to determine whether these methods were capable of consistently detecting KPC-mediated carbapenem resistance in fresh clinical isolates of *K. pneumoniae*.

## Materials and Methods

### Bacterial Isolates

To achieve a diversity of β-lactam resistance phenotypes, we selected 15 isolates of *K. pneumoniae* from 4 hospitals in New York City, all serviced by a central microbiology laboratory, on the basis of varying susceptibility patterns to imipenem, meropenem, and extended-spectrum cephalosporins. The isolates were from a variety of body sites (blood, sputum, and urine) and obtained in a 1-month period in 2005. The carbapenem-resistant quality control isolates *K. pneumoniae* 1534 (containing the KPC-1 carbapenemase) and *Serratia marcescens* 525 (which contains an SME-like β-lactamase) were from the Project ICARE (Intensive Care Antimicrobial Resistance Epidemiology) strain collection (1,11). The imipenem MICs for the isolates and the study identification of their hospital of origin are shown in Table 1.

**Table 1:** Carbapenem susceptibility and strain typing results for isolates tested in the study*

Organism	Imipenem broth microdilution MIC† (μg/mL) and CLSI interpretation	Meropenem broth microdilution MIC (μg/mL) and CLSI interpretation	PFGE profile	Hospital identification no.
1 *Klebsiella pneumoniae*	8 I	16 R	A	1
2 *K. pneumoniae*	16 R	>16 R	A	1
3 *K. pneumoniae*	16 R	16 R	A	2
4 *K. pneumoniae*	8 I	8 I	A	2
5 *K. pneumoniae*	16 R	>16 R	A	2
6 *K. pneumoniae*	16 R	16 R	A	2
7 *K. pneumoniae*	16 R	16 R	A	3
8 *K. pneumoniae*	32 R	16 R	A	4
9 *K. pneumoniae*	16 R	>16 R	B	1
10 *K. pneumoniae*	16 R	>16 R	B	1
11 *K. pneumoniae*	16 R	16 R	C	4
12 *K. pneumoniae*	16 R	16 R	D	1
13 *K. pneumoniae*	16 R	16 R	E	4
14 *K. pneumoniae*	16 R	>16 R	F	1
15 *K. pneumoniae*	16 R	>16 R	F1	4
*K. pneumoniae* 1534 (control)	16 R	>16 R	NA	NA
*Serratia marcescens* 525 (control)	>16 R	>16 R	NA	NA

*CLSI, Clinical and Laboratory Standards Institute; PFGE, pulsed-field gel electrophoresis; NA, not applicable.†Interpretations of MIC results used CLSI criteria (18); I, intermediate; R, resistant.

### Reference Susceptibility Testing Methods

The antimicrobial susceptibility profiles of the isolates were determined by the broth microdilution method with cation-adjusted Mueller-Hinton broth (BD Diagnostic Systems [BDDS], Sparks, MD, USA), as described in the National Committee for Clinical Laboratory Standards (NCCLS, Wayne, PA, USA) (now known as the Clinical and Laboratory Standards Institute [CLSI]) publication M7-A6 (16). Disk diffusion was performed as described in NCCLS document M2-A8 (17). Interpretations of MIC and disk diffusion results were made by using CLSI document M100-S15 (18).

### Commercial Susceptibility Testing and Molecular Methods

The Etest method (AB Biodisk, Solna, Sweden) was performed as described by the manufacturer with Mueller-Hinton agar (BDDS); tests were interpreted at 18–20 h. The MicroScan WalkAway (Dade MicroScan, Inc.), BD Phoenix (BDDS), Sensititre AutoReader (Westlake, OH, USA), VITEK Legacy (bioMérieux), and VITEK 2 (bioMérieux) systems were tested according to manufacturers' protocols. The panels and cards used are listed in Table 2. All systems were tested with inocula from the same subculture. All strains for which the commercial MIC results were discrepant with MIC results from the broth microdilution reference method were retested by using all methods.

**Table 2:** Summary of antimicrobial susceptibility testing results for 15 test isolates*

Method (software)	Card/panel	Imipenem results (n = 15)			Meropenem results (n = 15)		
		Resistant	Intermediate	Susceptible	Resistant	Intermediate	Susceptible
Broth microdilution	In-house frozen panel	13	2	0	14	1	0
Disk diffusion	BDDS disks	3	11	1	10	5	0
MicroScan (LabPro1.51, Alert 1.50)	Neg combo 32	7	7	1	13	1	1
Phoenix (4.05W/3.81A)	NMIC/ ID-104	5	8	2	12	1	2
Sensititre AutoReader (3.0.8 SP2)	GN2F	0	2	13	0	3	12
VITEK (R10.01)	Superflex GNS 122 and 127	5	0	10	2	3	10
VITEK 2* (R04.01)	GN07	4	6	5	4	4	5

*No meropenem interpretations were given by the Advanced Expert System for 2 organisms.

The carbapenem inactivation assay was performed on Mueller-Hinton agar with imipenem and meropenem disks as described by Yigit et al. (1). Isoelectric focusing was performed on crude lysates of isolates as previously described (1,2).

For detection of *bla*_KPC_ genes, a 489-bp internal gene fragment was amplified by using forward (5´-CTTGCTGCCGCTGTGCTG-3´) and reverse (5´-GCAGGTTCCGGTTTTGTCTC-3´) oligonucleotide primers where the 5´ base of each primer corresponds to position 223 or 711, respectively, with regard to the translational start site (*bla*_KPC-1_ numbering, GenBank accession no. AF297554). PCR reagents included a final concentration of 0.5 μmol/L of each primer and 2 mmol/L MgCl_2_. An annealing temperature of 60°C was used for amplification.

For DNA sequence determination, a 989-bp PCR product that included the entire *bla*_KPC_ structural gene was amplified by using oligonucleotide primers as previously described (4). Products were purified on QIAquick spin columns (Qiagen, Chatsworth, CA, USA). The nucleotide sequences of both strands of the *bla*_KPC_ gene from isolates 4 and 11 were determined from independent amplification products by using previously described primers (1). Cycle sequencing reactions were performed in a GeneAmp PCR system 9700 thermal cycler with the ABI BigDye Terminator v3.1 cycle sequencing kit (Perkin-Elmer, Applied Biosystems Division, Foster City, CA, USA). Products from sequencing reactions were purified on Cetri-Sep spin columns (Princeton Separations, Adelphia, NJ, USA) before analysis on an ABI 3130xl Genetic Analyzer. DNA sequencing data were analyzed by using DNASIS for Windows (Hitachi Software Genetic Systems, San Francisco, CA, USA).

## Results

### Carbapenem Testing with VITEK Legacy

Initial imipenem and meropenem susceptibility test results for 15 isolates of *K. pneumoniae* tested with the VITEK Legacy system with GNS 122 and 127 panels (flex system) in 1 hospital laboratory in New York City in a 1-month period in 2005 yielded a range of imipenem and meropenem MICs from susceptible (MIC <4 μg/mL) to resistant (MIC >16 μg/mL) (data not shown). One isolate was imipenem resistant (MIC >16 μg/mL) but meropenem susceptible (<4 μg/mL) on day 1, but on retesting the following day was meropenem resistant (16 μg/mL) and imipenem susceptible (<4 μg/mL). Imipenem Etest results, which were set up to arbitrate the conflicting results, were difficult to interpret because of variable numbers of colonies within the ellipses of inhibition (Figure). A carbapenem inactivation assay performed on 1 isolate indicated that it contained a carbapenem-inactivating enzyme (data not shown). The 15 isolates, which were collected from 4 different hospitals in New York City, were sent to the Centers for Disease Control and Prevention (Atlanta, GA, USA) for additional testing.

**Figure:**
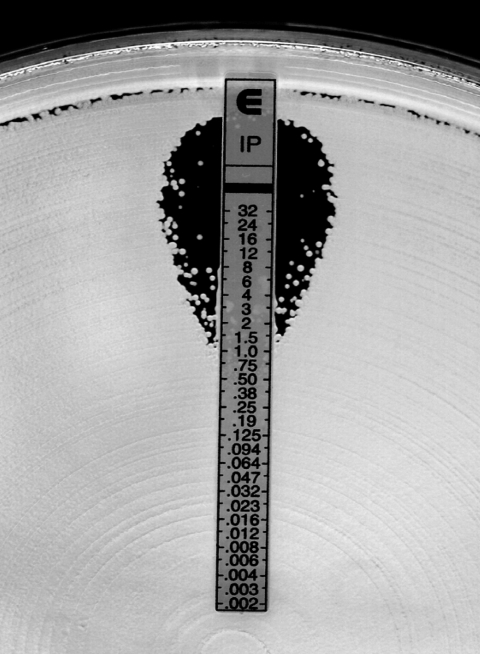
*Klebsiella pneumoniae* isolate tested with imipenem Etest strip (AB Biodisk, Solna, Sweden) on Mueller-Hinton agar. Inner colonies made determination of the imipenem MIC difficult.

### Characterization of *bla*_KPC_-containing *K. pneumoniae* Strains

By using the broth microdilution reference method, all the isolates were either intermediate or resistant to both imipenem and meropenem (Table 2). Two known imipenem-resistant isolates, *K. pneumoniae* 1534 and *S. marcescens* 525, were included as controls. Eight of 15 isolates from the 4 hospitals had the same pulsed-field gel electrophoresis (PFGE) type (data not shown), although their antibiograms varied for several antimicrobial agents (data not shown). Two isolates had a similar pattern but because of a 3-band difference were designated as type B. The remaining isolates showed patterns unrelated to types A or B (Table 1). All 15 isolates demonstrated 5 β-lactamase bands by isoelectric focusing (pIs = 5.4, 6.8, 7.0, 8.1, and 8.2), 1 of which was consistent with a KPC β-lactamase (pI = 6.8) (1,2). A 489-bp gene fragment was amplified from all 15 carbapenem-resistant *K. pneumoniae* isolates by using *bla*_KPC_-specific oligonucleotide primers. DNA sequence analysis of purified PCR products that included the entire coding region of the *bla*_KPC_ genes of isolates 4 and 11 (which had unique PFGE profiles) identified the β-lactamase genes as *bla*_KPC-2_ and *bla*_KPC-3_, respectively.

The 15 isolates were tested for imipenem and meropenem resistance by disk diffusion, Etest, MicroScan WalkAway, BD Phoenix, Sensititre AutoReader, VITEK, and VITEK 2 panels and cards. The results of testing are summarized in Table 2. MicroScan WalkAway reported 1 isolate as susceptible to both imipenem and meropenem, whereas the Phoenix system called 2 isolates susceptible to both imipenem and meropenem. VITEK called 7 isolates (representing 3 different PFGE profiles) susceptible to both imipenem and meropenem, 3 isolates resistant to imipenem but susceptible to meropenem, 1 isolate susceptible to imipenem but resistant to meropenem, and 2 isolates susceptible to imipenem but intermediate to meropenem. Of the final 2 isolates, 1 was resistant to imipenem and intermediate to meropenem, and the other was resistant to both antimicrobial agents. Thus, 10 (67%) of 15 isolates were interpreted on initial testing as susceptible to imipenem, and 10 were susceptible to meropenem. When the VITEK 2 system was used, 5 (33%) of 15 isolates were reported as susceptible to imipenem. The VITEK 2 Advanced Expert System (AES) used the imipenem results to predict meropenem results; thus, these same 5 isolates were called meropenem susceptible. In addition, as a result of the AES's recognizing unusual susceptibility results in the antibiograms of 2 *K. pneumoniae* isolates, AES did not report an interpretation for meropenem for 2 isolates. Finally, the VITEK 2 reported 2 isolates as imipenem resistant and 1 isolate as imipenem intermediate, although the MICs reported by AES were listed as <2 μg/mL. Aside from these, all AES categorical interpretations were in agreement with the original VITEK 2 MIC results.

When Sensititre panels were used, 10 (67%) of 15 isolates were reported as susceptible to both imipenem and meropenem, 2 isolates were reported as imipenem intermediate and meropenem susceptible, and 3 isolates were reported as imipenem susceptible and meropenem intermediate. Thus, on initial testing, 13 (87%) of 15 isolates were reported as imipenem susceptible, and 12 (80%) of 15 were meropenem susceptible. Repeat testing of strain 4 yielded imipenem- and meropenem-resistant results for MicroScan, BD Phoenix, VITEK Legacy, and VITEK 2. However, the Sensititre AutoReader results showed the isolate as susceptible to imipenem and meropenem. Retesting of strain 14 on the BD Phoenix showed the isolate as resistant to imipenem and meropenem. However, with VITEK Legacy, the isolate remained susceptible to imipenem, but the response to meropenem switched from 8 μg/mL (intermediate) to <4 μg/mL (susceptible), which confirmed the observations of flip-flopping (i.e., reversing) results from the New York City laboratory. The Sensititre AutoReader results for strain 14 remained susceptible on repeat testing; MicroScan results remained resistant.

## Discussion

Detecting KPC-mediated carbapenem resistance in *K. pneumoniae* isolates remains a challenge for many automated susceptibility testing systems. Although we used a total of only 17 isolates in this study (including 1 *S. marcescens* and 1 *K. pneumoniae* control), the isolates and controls represented 4 different carbapenemases (KPC-1, KPC-2, KPC-3, and an SME-like β-lactamase), 7 PFGE types, and variable imipenem and meropenem resistance profiles. Indeed, an important observation of this study is that the carbapenem-resistance profiles of the isolates varied from day to day, sometimes reversing from imipenem resistant/ meropenem susceptible to imipenem susceptible/meropenem resistant**.** Although in our study the MicroScan and BD Phoenix systems produced results that were more consistent with those with the reference testing systems than those with the VITEK and Sensititre AutoReader systems, problems detecting carbapenem resistance were still evident with the former systems. Bratu et al. suggested that part of the variability in detecting imipenem resistance with automated systems was a result of underinoculating the panels (14,15). Repeat testing of isolate 4 in our study with careful attention to inoculum appeared to improve results, which suggests that appropriate inoculum size is, indeed, a critical factor for achieving accurate results. The problem of the VITEK 2 AES reporting imipenem-resistant results as <2 μg/mL has apparently been corrected in software version R04.02.

Although we included the Etest method in our study, determining resistance and susceptibility for both imipenem and meropenem with Etest was difficult because colonies were present within the zones of inhibition. Since we could not achieve consensus on the interpretations among several readers who viewed the results, we did not include the Etest data in our analysis. Ertapenem Etest strips and disks were not tested in this study. This lack of consensus on reading Etest method, which is often used as a secondary testing method to confirm questionable results generated by automated methods, raises the question of which, if any, of the methods are reliable enough to be used for confirmation testing of carbapenem nonsusceptibility, particularly in *K. pneumoniae* isolates. Our data suggest that disk diffusion, especially with meropenem disks, may be used to confirm a carbapenem nonsusceptible result in *K. pneumoniae* isolates, which would warrant further testing. Whether this recommendation will hold true for other species of *Enterobacteriaceae* will require further study. Our data also suggest that if the interpretations of MIC or disk diffusion results for imipenem and meropenem for *K. pneumoniae* are discrepant, isolates should be retested with particular attention to using an adequate inoculum size. If treatment failure with carbapenems is observed for isolates of *K. pneumoniae* that were previously reported as susceptible to carbapenems, repeat testing with a nonautomated method, such as disk diffusion, may be warranted.
